# Changes in alcohol purchases from grocery stores after authorising the sale of stronger beverages: The case of the Finnish alcohol legislation reform in 2018

**DOI:** 10.1177/14550725221082364

**Published:** 2022-05-16

**Authors:** Liisa Uusitalo, Jaakko Nevalainen, Ossi Rahkonen, Maijaliisa Erkkola, Hannu Saarijärvi, Mikael Fogelholm, Tomi Lintonen

**Affiliations:** 3835 University of Helsinki, Finland; 7840 Tampere University, Finland; 3835 University of Helsinki, Finland; 3835 University of Helsinki, Finland; 7840 Tampere University, Finland; 98742 University of Helsinki, Finland; Finnish Foundation for Alcohol Studies c/o THL, Helsinki, Finland

**Keywords:** availability, beer, loyalty card data, price, ready-to-drink beverage, socio-demographic groups

## Abstract

**Aims:** The Finnish alcohol law was reformed in January 2018. The availability of alcoholic beverages in grocery stores increased as the legal limit for retail sales of alcoholic drinks was raised from 4.7% to 5.5% alcohol, and the requirement of production by fermentation was abolished. We analysed how the inclusion of strong beers, ciders, and ready-to-drink beverages in grocery stores was reflected in alcohol purchases, and how these changes differed by age, sex, level of education and household income. **Design:** The study sample included 47,066 loyalty card holders from the largest food retailer in Finland. The data consisted of longitudinal, individual-level information on alcohol purchases from grocery stores, covering the time period between 1 January 2017 and 31 December 2018. The volumes of absolute alcohol during a calendar year from beers, ciders, ready-to-drink beverages, and in total, were calculated. Alcohol purchases in 2017 and 2018 were compared. **Results:** There was no overall change in the total alcohol (0.04 [95% CI −0.03, 0.11] litres/year) or beer purchases (−0.05 [95% CI −0.11, 0.02] litres/year). Purchases of ready-to-drink beverages increased by 0.10 [95% CI 0.09, 0.11] litres/year (+ 84%). Total alcohol purchases increased in the three highest income groups, whereas they decreased in the two lowest groups (*p* for the interaction < 0.0001). **Conclusions:** The increased purchases of alcohol as ready-to-drink beverages were, on the average, compensated for by a decrease in purchases of other alcoholic beverages. Higher prices probably limited the purchases among lower income groups and younger consumers, while the increase was sharper in higher income groups.

Alcohol consumption in populations is affected by policy measures related to price and availability (Anderson et al., 2009; Gilmore et al., 2016). Total consumption tends to be lower in countries with strict alcohol policies (Brand et al., 2007), and changes in alcohol legislation influence consumption (Babor et al., 2003; Bruun et al., 1975). Finland had a state monopoly (Alko Inc.) comprising production, import, export and retail sale of alcohol from 1932, with the exception of the liberalization of medium strength beer (≤ 4.7%) to grocery stores from 1969 onwards (Mäkelä et al., 1981). When Finland joined the EU in 1995, the monopoly on alcohol imports, exports, production and wholesale was abolished (Alcohol Act, 1344/1994). Spirits and wines were retained under a retail sales monopoly, but milder beverages were also released to off-premises sales in grocery stores, kiosks and petrol stations. Total alcohol consumption increased by 12% during 1995 (Karlsson, 2018)*.* Today the off-premises purchases of mild alcoholic beverages are highly concentrated to supermarket-type stores. In 2017, the volume of ready-to-drink alcoholic beverages (RTD) sold in Alko Inc. was 25.2% of that in other off-premises outlets, while for beer the corresponding figure was only 3.3% (Valvira, 2019).

The Finnish alcohol law was reformed at the beginning of 2018 (Ministry of Social Affairs and Health, 2018). Two important relaxations were implemented. First, the legal limit for beverages sold in grocery stores was raised from ≤ 4.7% to ≤ 5.5% alcohol by volume. Second, the requirement that alcohol sold in grocery stores had to be produced by fermentation was abolished. Hence, the availability of alcoholic beverages in grocery stores increased in January 2018 with the inclusion of stronger beers, ciders and RTD beverages mixed from distilled spirits in the selections. The Finnish Institute for Health and Welfare expected that the prices of these beverages would decrease compared with their prices in alcohol monopoly stores (Mäkelä & Österberg, 2017). In addition, the number of outlets selling these beverages increased 15-fold (Mäkelä & Österberg, 2017). At the same time, the alcohol tax was raised, leading to an increase of around 5% in the retail prices of alcoholic beverages (Ministry of Finance, 2017).

The national statistics on alcohol sales indicate a general decreasing trend in alcohol consumption in Finland since 2007 (Jääskeläinen & Virtanen, 2020). From 2016 to 2017, a year before the law reform, there were minor decreases in both retail (off-premises) and total (off- and on-premises) sales (Valvira*,* 2019). Due to the changes in availability and price, the national authorities predicted an increase in population alcohol consumption after the introduction of the new alcohol law (Mäkelä & Österberg, 2017). However, the statistics revealed no overall increase in alcohol sales from 2017 to 2018 (Jääskeläinen & Virtanen, 2019). There was a shift from monopoly stores to other off-premises stores in 2018 compared with 2017. Sales (measured as 100% alcohol) in monopoly stores decreased by 5.1%, whereas in grocery stores and other off-premises sales points of sale they increased by 4.6% (Valvira, 2019). A larger proportion of 100% alcohol was sold as stronger varieties (≥ 4.7% alcohol) in 2018 compared to 2017. For RTD beverages, this proportion increased from 35.4% to 72.5%, for beer from 6.2% to 14.4%, and for cider from 4.5% to 8.5% (Jääskeläinen & Virtanen, 2019).

The effects of alcohol policies may vary in different population subgroups. It is well documented that increases in prices lead to reduced consumption (Sharma et al., 2017), while the magnitude of price elasticity differs by socio-demographic factors. The evidence on the effect of price in different age groups is inconsistent (Chaloupka et al., 2002; Giesbrecht et al., 2016; Helakorpi et al., 2010; Sharma et al., 2017). Young people have been reported as being more (Chaloupka et al., 2002; Giesbrecht et al., 2016) or less (Sharma et al., 2017) responsive to price changes. After the alcohol tax reduction in Finland in 2004, moderate and heavy drinking increased among those aged ≥ 45 years old, but not among younger age groups (Helakorpi et al., 2010). Little is known as yet about responses according to socio-economic status (Giesbrecht et al., 2016). After the Finnish tax reduction in 2004, alcohol consumption increased in lower educated groups in particular (Helakorpi et al., 2010). Alcohol-related deaths increased among people aged ≥ 35 years who had a low income, a low education level, or who were unemployed (Herttua et al., 2008). Availability has been studied for the most part in terms of hours and days of sale and density of alcohol outlets (Anderson et al., 2009; Gilmore et al., 2016; Gmel et al., 2016; Popova et al., 2009; Sherk et al., 2018; Siegfried & Parry, 2019), while little is known about how changes in variety within outlets affect alcohol purchases. The increased availability of RTD beverages may be expected to appeal to young people in particular (Fortunato et al., 2014).

In order to enhance understanding of the linkage between alcohol legislation and consumption, this study focuses on exploring the effects of recent changes in Finnish alcohol legislation. Specifically, the aim of the study was to analyse how the inclusion of strong beers, RTD beverages and ciders in grocery stores was reflected in alcohol purchases after the new law was enacted on 1 January 2018. While overall consumption trends are monitored by national statistics, our exceptionally large purchase data with detailed individual-level longitudinal information demonstrated how the changes in purchases differed by age, sex, level of education and household income.

## Methods

### Study participants

The data used in the current study consisted of longitudinal, individual-level information on alcohol purchases. The data was obtained from the S Group, which is the largest food retailer in Finland with a market share of approximately 46% (Finnish Grocery Trade Association, n.d.; S Group, n.d.). By registering their purchases with a loyalty card at the cash desk, the card holder earns a small financial reward for grocery purchases (albeit not allowed for alcoholic beverages since 1 March 2018). Having a loyalty card is optional, and purchases can be made without holding or using a loyalty card. A household can use a shared loyalty account with one person defined as the primary card holder. Full details of the data collection were described in Vuorinen et al. (2020).

The study participants were recruited across Finland by an email sent by the S Group in June 2018. The primary card holder of each loyalty account was asked for their consent to release their grocery purchase data for research purposes, and requested to complete a voluntary online questionnaire. The email was sent to > 1.1 M primary card holders who had submitted their email addresses to the retailer’s database, were aged ≥ 18 years old, and had not refused contact for marketing or research purposes (58% of all card holders), of whom 47,066 consented. It was not possible to calculate a reliable participation rate, because we had no information on how many card holders were actually reached (Vuorinen et al., 2020). We do not know how many email addressed were valid, passed through spam filters, or were ignored by the recipient as advertisement. The online questionnaire was completed by 36,621 card holders (78% of those who consented). The data used in this study covered the time period between 1 January 2017 and 31 December 2018.

### Measurement of alcohol purchases

In our earlier study analysing the agreement between the purchase data used in this study and independent measurement by a frequency questionnaire completed by the same participants, we showed that information on beer purchase days derived from the loyalty card database can be used to estimate beer drinking frequency with fair to good accuracy (Lintonen et al., 2020). We used the volume of absolute alcohol (in litres) from beers, ciders, RTD beverages, and the total absolute alcohol as the outcome variables. For all purchased items, the data contained an item description, time stamp, volume, and expenditure (in euros). Alcoholic beverages were grouped in terms of beverage type (beer, cider, RTD beverage or wine), volume, and alcoholic concentration (≤ 1.2%, 1.3–2.8%, 2.9–3.5%, 3.6–4.7%, and 4.8–5.5%). We did not analyse wine purchases in this study. Only mild wines with an alcohol concentration of ≤ 5.5% are sold in Finnish grocery stores, and wine is mainly purchased from state alcohol monopoly stores. In our data, the share of wine was only 0.03% of total purchases of absolute alcohol in 2018. We calculated the volumes of absolute alcohol for each shopping occasion by multiplying the volume of each item by its alcohol concentration and by adding these up across beverage type (beer, cider, RTD beverage, total). Unless otherwise stated, by price of alcohol we mean the price of alcohol actually purchased by each card holder, instead of the prices of products offered at grocery stores.

### Socio-demographic variables

Sex, age, level of education, and household income were analysed as determinants of alcohol purchases. Information on birth year and sex were obtained from the retailer’s database. Age was calculated from the birth year and categorised into age groups: 18–29, 30–44, 45–59, and ≥ 60 years. Information on the level of education of the respondent and on the monthly gross income of the household was requested in the online study questionnaire. Household income was scaled to the size of the household by dividing income by the square root of household size (OECD Project on Income Distribution and Poverty, n.d.), and categorised into groups of < 1000, 1000–1999, 2000–2999, 3000–3999, and ≥ 4000 €/month.

### Statistical methods

The time series of purchased alcohol by beverage type were constructed by computing the daily totals across all individual card holders. We applied a classical decomposition method to extract the seasonal weekday component, assuming a multiplicative model. We then adjusted the time series by removing it. Each de-seasonalised daily total was compared to the same date of the previous year and the differences between the two were plotted, as well as the 30-day moving average of the differences. The distribution of annual volumes, expenditure and price of purchased alcohol by beverage type and beverage alcohol concentration were presented using descriptive statistics.

The main comparison of the before−after setting was analysed using the annual totals of purchased alcohol per card holder, overall and by beverage type. The analysis was performed with repeated measurements analysis of variance. Differences in changes between population subgroups (sex, age group, level of education, and household income) were identified by the incorporation of the corresponding factor and factor by time interaction into the model, and the statistical significance (5% type 1 error rate) of the latter.

Loyalty card data provide objective measures on alcohol purchases that could decrease information biases, but participants generating the data could be a highly selected subgroup. In a recent publication, we have assessed the representativeness of our study sample, and constructed weights by using background information given by the card holders in order to match their socio-demographic distributions with the Finnish population distributions as closely as possible (Vuorinen et al., 2020). In the present study, the main analyses were weighted to improve the representativeness of the sample to the adult Finnish population.

The analyses included all consenting individuals for whom weights could be computed (Vuorinen et al., 2020), and the analyses of the population subgroups of those consenting individuals who had provided information on the respective factor.

### Ethical aspects

The study was approved by the University of Helsinki Review Board in the humanities and social and behavioural sciences (Statement 21/2018). Informed consent was collected electronically from all participants included in the study when they were invited, by email, to release their loyalty card data and complete the background questionnaire. The S Group pseudonymised the data before transferring it to the research group.

The data used in this study are owned by a third party (S Group) and were used under a research agreement for the current study, and are not publicly available. According to the research agreement, the authors are not allowed to share the data.

## Results

### Participant characteristics

The characteristics of the participating loyalty card holders (*n* = 47,066) are presented in Table 1. The mean age was 47 years, and two-thirds were women. A fifth of the participants had completed a master’s degree or higher.

**Table 1.** Characteristics of the participating loyalty card holders of a large Finnish retail chain (*n* = 47,066).

Background characteristic	*n*	%
Sex		
Male	16,349	34.70
Female	30,696	65.20
Missing	21	0.04
Age group		
18−29 years	5,846	12.40
30−44 years	15,048	32.00
45−59 years	13,764	29.20
60+ years	12,387	26.30
Missing	21	0.04
Education level		
Primary school or less	2,268	4.80
Upper secondary school	13,481	28.60
Lower degree-level tertiary education	11,792	25.10
Higher degree-level tertiary education	8,911	18.90
Other	17	0.04
Missing*	10,597	22.50
Household income (€/month), scaled for household size		
<1000	3,201	6.80
1000−1999	5,352	11.40
2000−2999	10,500	22.30
3000−3999	8,089	17.20
≥4000	6,833	14.50
Missing*	13,091	27.80

*Did not provide an answer in the questionnaire.

### Changes in alcohol purchases overall and by beverage type

Alcohol volume, expenditure, and price of purchases by study year and type of beverage are presented in Table 2. Beer was by far the most popular type of alcoholic beverage purchased from the target grocery stores.

**Table 2.** Price, expenditure and volume of purchased alcohol among loyalty card holders of a large Finnish retail chain during 2017 and 2018 (weighted to improve representativeness of the sample to the adult Finnish population).

	Mean (*SD*)					
	Price of purchased alcohol (€/l alcohol)		Total alcohol expenditure (€)		Volume of 100% alcohol (l)	
Type of beverage	2017	2018	2017	2018	2017	2018
Beer	81.4 (28.8)	87.2 (29.1)	107.3 (294.7)	113.8 (293.3)	1.7 (5.1)	1.6 (4.6)
Cider	128.2 (33.6)	132.1 (28.0)	18.3 (89.1)	17.1 (80.2)	0.2 (0.9)	0.1 (0.7)
Ready-to-drink	112.3 (24.2)	132.8 (48.0)	12.4 (58.3)	26.9 (92.3)	0.1 (0.7)	0.2 (0.9)
Total	92.0 (37.0)	100.9 (39.0)	138.1 (338.1)	158.0 (351.8)	1.9 (5.4)	2.0 (5.1)

**Figure 1.**
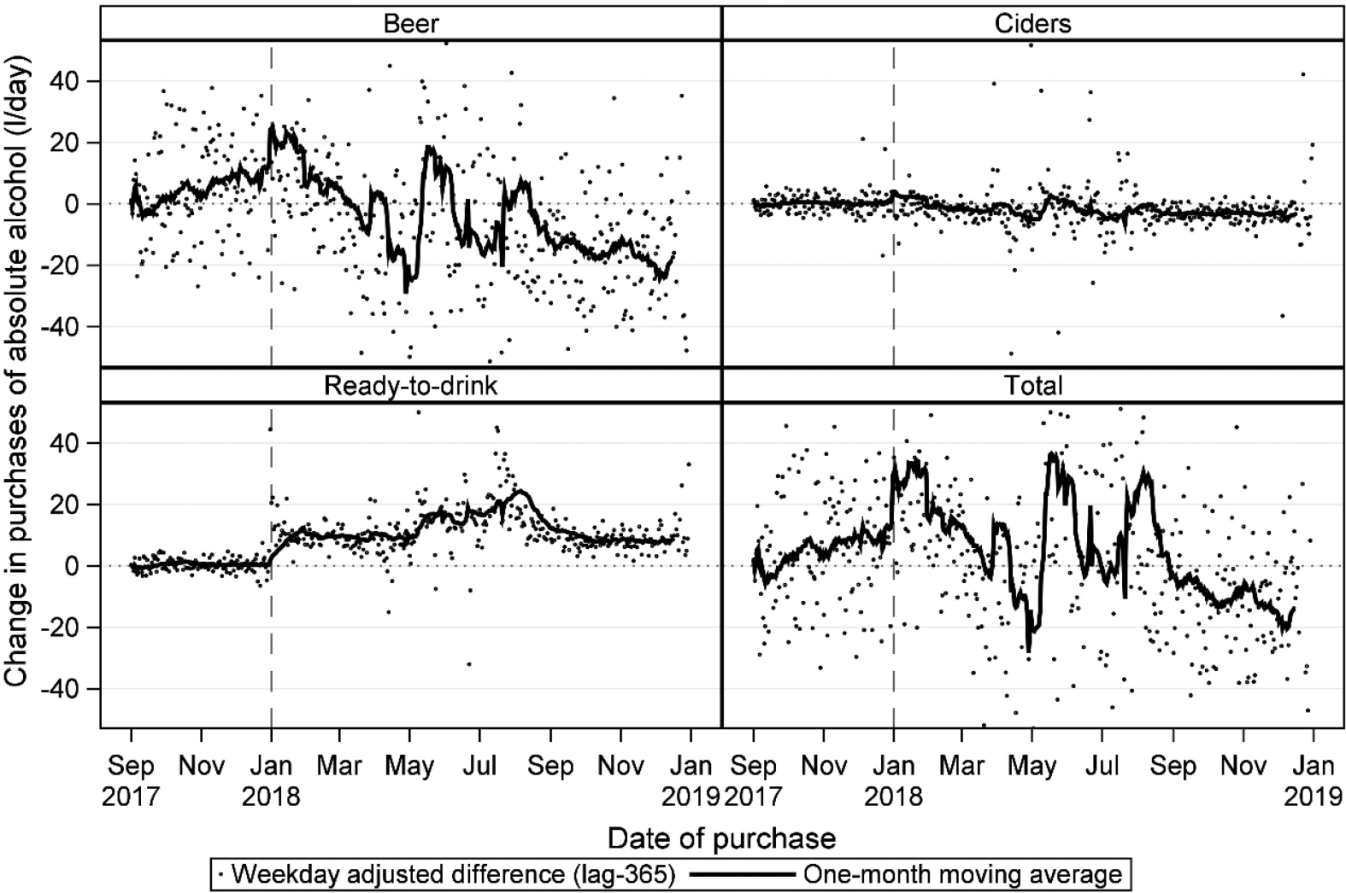
Weekday adjusted 365-day difference in purchases of beer, cider, ready-to-drink alcoholic beverages and total alcoholic beverages, measured as absolute alcohol, between the years 2017 and 2018 among loyalty card holders of a large Finnish retail chain.

The changes from 2017 to 2018 by beverage type are depicted in Figure 1, which shows the difference in purchased volume compared with the same date one year earlier. There was no overall change in the total amount of absolute alcohol (0.04 [95% CI −0.03, 0.11] litres/year) or in the alcohol purchased as beer (−0.05 [95% CI −0.11, 0.02] litres/year). Alcohol from cider purchases decreased from 2017 to 2018 by 0.02 [95% CI 0.01, 0.03] litres of absolute alcohol/year (−10%), whereas RTD purchases increased by 0.10 [95% CI 0.09, 0.11] litres of absolute alcohol/year ( + 84%).

### Price of alcohol and alcohol expenditure

The price per unit of alcohol purchased as stronger alcoholic beverages (4.8–5.5% alcohol) was, on average, considerably higher (130.8 euros/litre) than that purchased as milder beverages in 2018 (96.1 euros/litre). The mean prices paid by the participants for their alcohol purchases rose slightly from 2017 to 2018 for all beverage types (Table 2). The increase was most pronounced for RTD beverages (+ 18%). The mean annual expenditure on RTD beverages more than doubled from 2017 to 2018, and also rose for beer and total alcoholic beverages, whereas the expenditure on cider decreased by 6%. Moreover, when considering the alcoholic beverages available in grocery stores, the average prices of stronger alcoholic beverages were higher than those of milder varieties (data not shown).

### Changes in alcohol purchases by socio-demographic group

**Figure 2.**
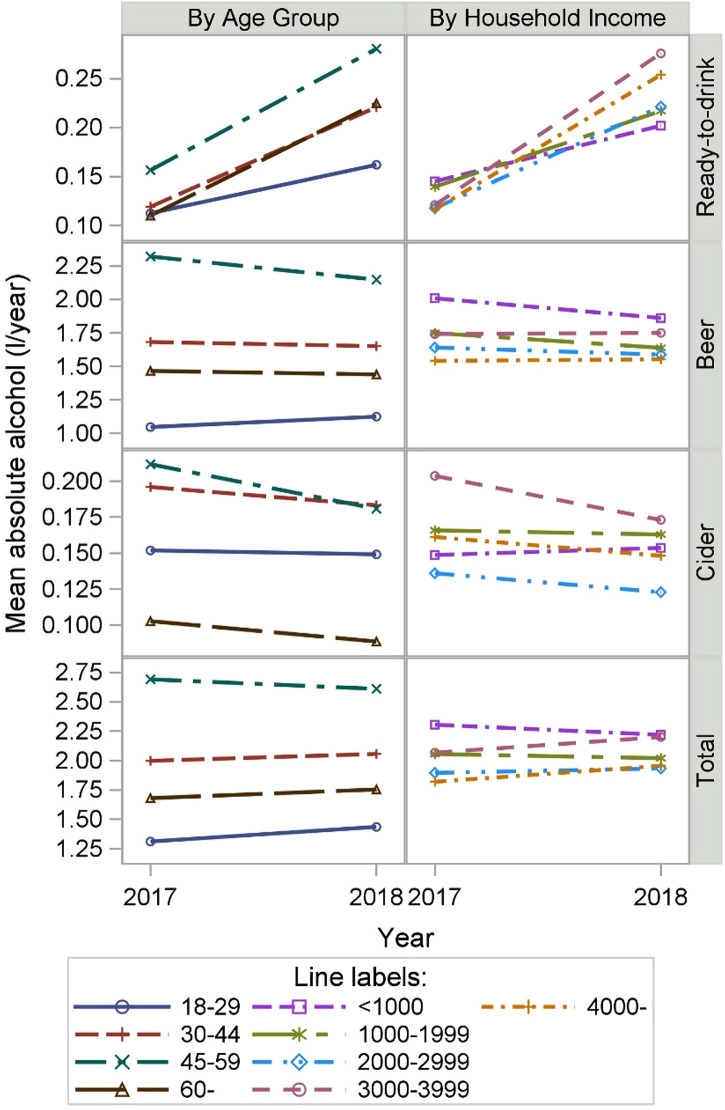
Change in purchases of beer, cider, ready-to-drink alcoholic beverages and total alcoholic beverages, measured as absolute alcohol, between 2017 and 2018 according to household income and age group among loyalty card holders of a large Finnish retail chain (weighted to improve representativeness of the sample to the adult Finnish population).

Those in later middle age (45–59 years) purchased the largest amounts of alcohol in both study years (Figure 2). The changes in the purchased volumes of absolute alcohol from 2017 to 2018 differed between age groups (*p* < 0.0001 for the interaction test) (all beverage types and total). Compared with 2017, the total amounts of alcohol purchased by different age groups remained in the same order, but the differences between the age groups had narrowed somewhat. Purchases of RTD beverages increased in all age groups, but the increase was smallest among the youngest age group of 18- to 29-year-olds (Figure 2). The increase in RTD purchases was largest among 45- to 59-year-olds, with an increase of 0.12 litres of absolute alcohol from 2017 to 2018. Absolute alcohol from beer and cider tended to decrease or to remain at the same level, with the exception of a small increase in beer purchases in the youngest age group.

Men purchased more alcohol in total and as beer and RTD beverages, and less alcohol as cider than women in both study years (Figure S1, online supplemental material). The sex-specific changes in purchases between 2017 and 2018 were similar to the overall changes (no noticeable change in total alcohol and beer, increase in RTD, decrease in cider). However, the increase in RTD purchases, and the decrease in cider purchases, was more pronounced among men (Figure S1, online supplemental material).

The level of education was inversely associated with total alcohol, beer and RTD purchases in both study years, while cider was purchased the most in the two middle education groups (Figure S1, online supplemental material). The changes in purchases between 2017 and 2018 were similar to the overall changes for all education groups.

**Figure 3.**
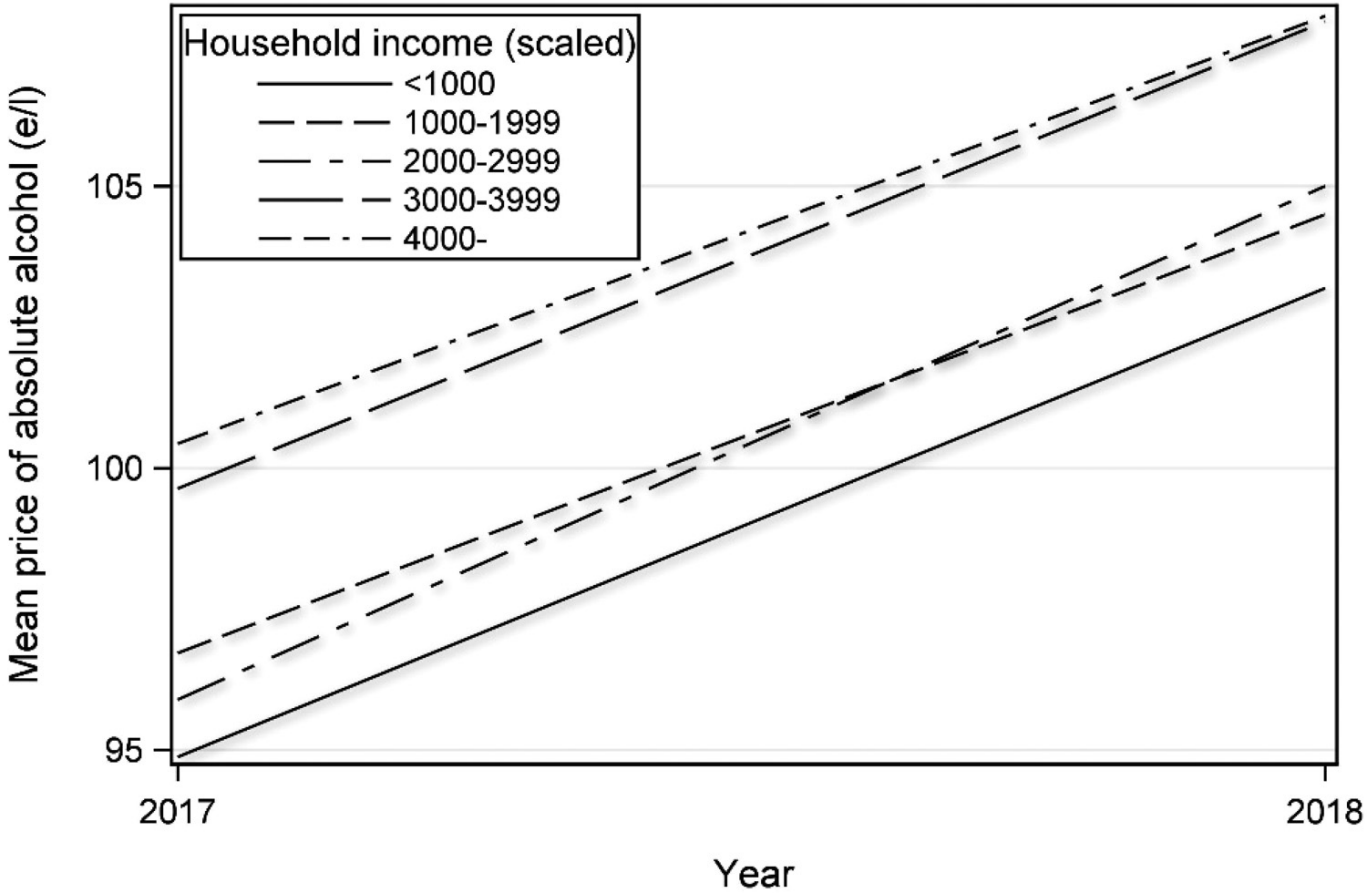
Mean price of alcoholic beverages (€/l absolute alcohol) by household income scaled according to household size (€/month) purchased by the loyalty card holders of a large Finnish retail chain during the study years 2017 and 2018 (weighted to improve representativeness of the sample to the adult Finnish population).

Non-parallel changes in alcohol purchases from 2017 to 2018 were observed between income groups (Figure 2). Total purchases increased in the three highest income groups, whereas they decreased in the two lowest groups (*p* for the interaction < 0.0001). The order of income groups according to RTD purchases changed, as the purchased amount of RTD beverages in the three highest income groups rose above that in the two lowest income groups. There was a decrease in beer purchases, especially among the two lowest income groups, with no meaningful change in the two highest income groups (*p* for the interaction < 0.0009). The higher the household income, the higher the average price of the alcohol purchased (Figure 3). Similarly, the total alcohol expenditure between 2017 and 2018 increased along with an increase in income (Table S2, online supplemental material). The growth in expenditure was most pronounced for RTD beverages, more than doubling in the three highest income groups.

Socio-demographic group analyses were also performed separately for low-strength and newly available strong (4.8% to 5.5% alcohol content) beer. Figures before and after the law change for low strength beer were studied as well as alcohol purchased in the form of strong beers.

Total alcohol purchases of beer in terms of 100% alcohol remained the same in 2018 (1.61 litres) compared with 2017 (1.66 litres). In 2018, 6% of beer in 100% alcohol was purchased in the form of newly available strong beer (alcohol content 4.8% to 5.5%; Table 3).

Table 3. Average volumes and standard deviations of <=4.7% and >4.7% beer in litres of 100% alcohol before and after the alcohol law change.

**Table 3.** 

	Vol 100% alcohol (l)	
	2017	2018
Beer <= 4.7%	1.66 (SD 5.05)	1.51 (SD 4.57)
Beer >4.7%	0.00 (SD 0.00)	0.10 (SD 0.47)

Decreases were seen among both sexes in purchases of <=4.7% beer, but the decrease was nearly five-fold among men (p<0.001 for the gender difference in the change). Changes were also different between age groups (p<0.001): no change in purchases of <=4.7% beer was seen in the group 18 to 29 years of age, but statistically highly significant decreases were observed among 30 to 44, 45 to 59 and those 60 years of age or older. Decreases in purchases of <=4.7% beer were seen at all educational levels, and the decreases did not differ significantly (p=0.107). Low strength beer (<=4.7%) purchases decreased in all income groups; there were no differences in the decreases between income groups (p=0.185) (Table 4).

Addendum Table 4. Changes in 100% alcohol (litres) from <=4.7% and >4.7% beer, and all beer from 2017 to 2018 by income group.

**Table 4.** 

Income groups	beer <=4.7%	95% CI	beer >4.7%	95% CI	beer total
0–1000	−0.20	(−0.27; −0.13)	0.05	(0.04; 0.07)	−0.15
1000–2000	−0.18	(−0.23; −0.12)	0.07	(0.06; 0.08)	−0.11
2000–3000	−0.15	(−0.20; −0.11)	0.10	(0.09; 0.11)	−0.05
3000–4000	−0.10	(−0.15; −0.04)	0.11	(0.10; 0.12)	+0.01
>=4000	−0.14	(−0.20; −0.04)	0.15	(0.13; 0.16)	+0.01

On average, men purchased three times as much >4.7% beer from S Group grocery stores as women (p<.001) after the legislation change in 2018. Purchases were, on average, higher among 30- to 59-year-olds than those aged 18 to 29, and highest among those aged 60 or older. No differences (p=0.060) were observed in purchases by educational level. Purchases of newly available >4.7% beer were higher in middle- and high-income groups than in the lower income groups (Table 4).

Total beer purchases in terms of volume of 100% alcohol decreased among those in the two lowest income groups but remained unchanged among the middle- and higher-income groups (Table 4). Part of the decrease in <=4.7% beer was compensated by purchases of newly available >4.7% beer. These purchases were dependent on household income of the buyer: the higher the income, the more purchases of >4.7% beer.

The average price per unit of alcohol purchased in the form of <=4.7% beer increased from 81.36 euros/litre in 2017 to 85.15 euros per litre in 2018. Alcohol in the form of strong beer was considerably more expensive: 116.82 euros per litre (available only in 2018).

## Discussion

Using a unique, large-scale purchase data, we examined how the law reform allowing the sale of stronger alcoholic beverages in grocery stores in Finland from 1 January 2018 was reflected in alcohol purchases measured as absolute alcohol among different socio-demographic groups. The overall alcohol purchases did not increase between 2017 and 2018. An increase in total alcohol purchases was observed only among those with a higher household income, while total alcohol purchases decreased among the lower income groups. The purchases of RTD beverages increased in all analysed population subgroups, but the increase was smallest in the youngest age group of 18- to 29-year-olds.

Despite the fact that an increase in total alcohol purchases was not observed in our study sample after the implementation of the alcohol law reform in January 2018, it cannot be concluded that the law reform had no effect on alcohol purchases. National statistics reveal a decreasing trend for alcohol consumption in Finland since 2007 (Jääskeläinen & Virtanen, 2020). In 2018, alcohol consumption remained at approximately the same level as in 2017 (Jääskeläinen & Virtanen, 2019), however, this was followed by another decrease in 2019 (Jääskeläinen & Virtanen, 2020). The levelling of consumption observed in 2018 coincides with the law reform and could have resulted from the changes it brought about. The effects of the law reform cannot be separated from other contemporary changes, however. For instance, the weather was exceptionally hot in Finland during the summer of 2018 with 64 hot days compared to 19 hot days in 2017 (Finnish Meteorological Institute, n.d. a, b), which may have led to an increased demand for mild alcoholic beverages and RTD alcoholic beverages in particular. Moreover, the purchasing power of wage and salary earners increased from 2017 to 2018, but this is an unlikely explanation for the levelling of alcohol consumption in 2018, because the same trend has prevailed concurrently with the decreasing trend of consumption (Statistics Finland, n.d. a, b).

In addition, we observed shifts between beverage types. The purchases of RTD beverages measured as absolute alcohol increased sharply after the law reform in 2018, whereas beer purchases did not change significantly, and cider purchases decreased slightly. In parallel with the law reform, retailers have been active in developing their product categories to better address consumer needs and preferences. For example, the availability of stronger beers from different local breweries in grocery stores’ product categories has been enhanced. In terms of product categories, the inclusion of stronger “long drinks” – a traditional Finnish RTD alcoholic beverage – affected the increase in the availability of RTD beverages.

Previous studies have shown that flavoured alcoholic beverages are very popular among young people (Fortunato et al., 2014). Surprisingly, the increase observed in RTD purchases among all age groups was most pronounced among middle-aged participants, and least pronounced among the youngest age group of 18- to 29-year-olds. According to earlier research, younger people tend to be more responsive to alcohol price fluctuations (Chaloupka et al., 2002; Giesbrecht et al., 2016; Giesbrecht et al., 2012), although this was not supported by a Finnish study on the effects of tax reduction (Helakorpi et al., 2010). The higher price of RTD beverages is a potential explanation for the more moderate increase in RTD purchases among the youngest age group observed in the present study.

While the changes related to alcoholic beverages from 2017 to 2018 were similar in different education groups, the changes differed according to household income. Total alcohol purchases increased among higher income groups, while they decreased among those with a lower income. Price issues are a likely explanation for the observed differences between the income groups. The strong alcoholic beverages have not been introduced as products on offer by the retailers (Mäkelä & Norström, 2019), and our data confirmed the higher average prices of stronger alcoholic beverages available in grocery stores compared with milder varieties. Alcohol in the form of strong beer was considerably more expensive than in the form of <=4.7% beer and, as a result, was favored by groups that were better able to afford them: the higher income groups. Alcohol tax was raised at the same time when the new law came into effect, and the prices of milder varieties also increased slightly. Price increases are effective means to control alcohol consumption (Sharma et al., 2017), although the degree of price elasticity varies between countries and time periods (Moskalewicz et al., 2016). In eastern European countries experiencing the political and economic upheaval beginning in the late 1980s, the relationship between alcohol affordability and consumption was clearly observed. For instance, in most countries, weakened state control allowed for lower taxes and increased alcoholic content of beer, leading to doubled consumption in less than two decades (Moskalewicz et al., 2016). While alcohol is taxed in more than 90% of countries, a few countries have set minimum prices for alcohol, reducing the availability of the cheapest alcoholic beverages (Sharma et al., 2017). According to evaluation studies in Canada (Stockwell et al., 2011) and Scotland (Robinson et al., 2021; Xhurxhi, 2020), minimum pricing effectively reduced alcohol consumption. Modelling studies suggest that minimum pricing affects especially high consumers (Sharma et al., 2017; van Walbeek & Chelwa, 2021), particularly those with lower income (Sharma et al., 2017).

We observed an increase in the mean price of purchased alcohol, and in expenditure on alcoholic beverages, especially RTD beverages, among all income groups. As could be expected based on previous research on price elasticities within income groups (Sharma et al., 2017), the shift was much more pronounced among those with a higher income. It was also reflected in an increase in total alcohol purchases in these groups. In contrast, a compensation effect was seen among lower income groups in that their beer purchases decreased along with increasing RTD purchases. A stronger effect of price on alcohol consumption among lower income groups has previously been observed (Jiang et al., 2016). In contrast, no systematic differences according to income level were observed after the reduction of the alcohol tax in Finland in 2004 (Mäkelä et al., 2008). As recent results on socio-demographic differences in Finland are lacking, it is not known whether the decreased purchases from grocery stores among lower income groups were compensated for by imported alcohol, for example. Travellers’ alcohol imports measured as absolute alcohol increased in 2018 by 3.8% (Karlsson & Jääskeläinen, 2019).

A vast amount of data was processed for the present study, with more than 47,000 participants from all over the country and a timeframe spanning two calendar years. The data include detailed purchase information on both the volume and the price of alcohol. In self-reported surveys, price information is difficult to measure, and potentially biased (Sharma et al., 2017). Scanner data has been suggested as the most accurate data type to analyse price elasticity (Ruhm et al., 2012). Our loyalty card data share the fine details of scanner data, but have the additional advantage of not being dependent on participant compliance and memory in scanning their purchases. The results of our recent validation study comparing data on alcohol purchases using a loyalty card (LoCard) with self-reported drinking frequency indicated a clear association between these methods (Lintonen et al., 2020). A limitation of the data is that they originate from a single retail chain, which is a potential source of bias if customer characteristics are associated with the choice of retailer. More importantly, we do not have information on study participants’ alcohol purchases from other retailers. However, the retail market is highly centralised in Finland, and the S Group – as the largest trade group – commanded a grocery market share of 46.4% in 2018 (Finnish Grocery Trade Association, n.d.). According to the S Group, 88% of Finnish households have registered purchases in their database. Based on the online survey conducted for this study, the degree of loyalty was quite high: 64% of participants reported that they had made 60% of their food purchases in the S Group’s stores (Vuorinen et al., 2020).

Compared to the general Finnish population, the employed, the middle-aged, women, and individuals with a higher education were over-represented in the sample, as was shown in our recent analysis (Vuorinen et al., 2020). We addressed the bias by using weights to match the socio-demographic distributions with the adult Finnish population distributions as closely as possible. Furthermore, it may well be that risky consumers of alcohol were less likely to agree to participate, and could therefore be under-represented in the study sample. As can be expected based on on-premises and monopoly sales and the market shares of other retail chains in Finland, the purchased amounts of alcohol in this study are lower than the national consumption figures. For example, the average purchase of beer was 1.6 litres as absolute alcohol in 2018, while the corresponding national statistic was 4.0 l per capita (Jääskeläinen & Virtanen, 2019). This may be partly explained by selection bias in the study sample, and partly by alcohol purchased from other on- or off-premises points of sale. However, the relative changes in the purchased total volumes (100% alcohol) of beverage types in our data are very similar to those reported in national statistics for off-premises stores, excluding monopoly stores (Valvira, 2019). The percentage changes in the present data versus national statistics were +84%/+76% for RTD beverages, −2.8%/−0.5% for beer, −10%/−9% for cider, and 2.2%/4.6% overall, respectively. This, together with the fact that the focus of our analysis is to compare data from the same individuals from two study years, adds credibility to our findings. The absolute strength of our data is that we were able to analyse purchases for different socio-demographic groups. This may be very important when following effects of changes in alcohol legislation and policies in general. These data cannot be obtained from national consumption statistics or store-based sales data. 

## Conclusions

In conclusion, in line with the Finnish statistics, no overall increase in total alcohol purchases was observed from 2017 to 2018. Considering the decreasing trend in alcohol consumption in Finland, the new alcohol law may still have contributed to the levelling of consumption observed in 2018. The results of this study can be interpreted as an interplay between availability and price as factors affecting alcohol purchases. In all population subgroups, there was interest towards the expanded selection of RTD beverages, but their higher price probably limited the purchased amounts among lower income groups and younger participants. The changes in alcohol purchase patterns after the law reform were similar according to sex and education level. In general, higher socio-economic status is associated with a healthier lifestyle and lower health risks (Mackenbach, 2006), but in the present case higher income was associated with unfavourable change in health behaviour in the form of increased alcohol purchases. RTD beverages have raised concern because they are seen as particularly appealing to young people, but based on our results, they are increasingly popular among all age groups. The information on socio-economic determinants of purchase behaviour in connection with changes in availability and price of alcoholic beverages can be used to guide future alcohol policy measures. On the basis of the present results, it seems important not to disregard the well-off adult population when planning alcohol policies.

## Supplemental Material

sj-docx-1-nad-10.1177_14550725221082364 - Supplemental material for Changes in alcohol purchases from grocery stores after authorising the sale of stronger beverages: The case of the Finnish alcohol legislation reform in 2018 Click here for additional data file. Supplemental material, sj-docx-1-nad-10.1177_14550725221082364 for Changes in alcohol purchases from grocery stores after authorising the sale of stronger beverages: The case of the Finnish alcohol legislation reform in 2018 by Liisa Uusitalo, Jaakko Nevalainen, Ossi Rahkonen, Maijaliisa Erkkola, Hannu Saarijärvi, Mikael Fogelholm and Tomi Lintonen in Nordic Studies on Alcohol and Drugs
