# Supplementary material for: Changes in alcohol purchases from grocery stores after authorising the sale of stronger beverages: The case of the Finnish alcohol legislation reform in 2018
Source: Nordisk Alkohol Nark. 2022 May 16;39(6):589–604. doi: 10.1177/14550725221082364 (PMC9703366; doi:10.1177/14550725221082364)
Supplement: sj-docx-1-nad-10.1177_14550725221082364 - Supplemental material for Changes in alcohol purchases from grocery stores after authorising the sale of stronger beverages: The case of the Finnish alcohol legislation reform in 2018 [file sj-docx-1-nad-10.1177_14550725221082364.docx]

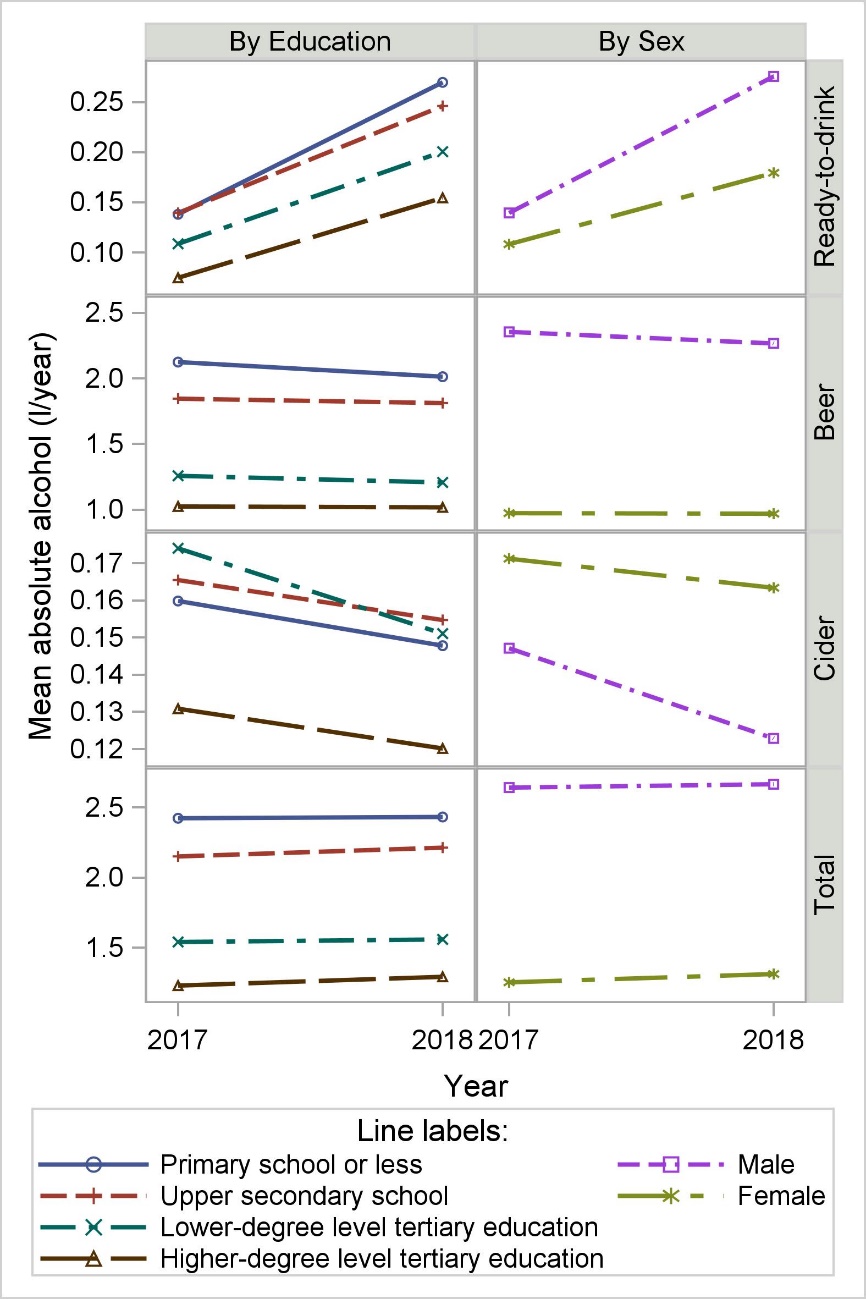


Figure S1. Change in purchases of beer, cider, ready-to-drink alcoholic beverages and total alcoholic beverages, measured as absolute alcohol, between 2017 and 2018 according to level of education^1^ and sex^2^ among loyalty card-holders of a large Finnish retail chain (weighted to improve representativeness of the sample to the adult Finnish population).

^1^*p* for interaction between education groups: 0.3365 for total, 0.048 for beer, <0.0001 for ready-to-drink beverages, and 0.2097 for cider

^2^ *p* for interaction between sexes: 0.128 for total, <0.0001 for beer, ready-to-drink beverages, and cider


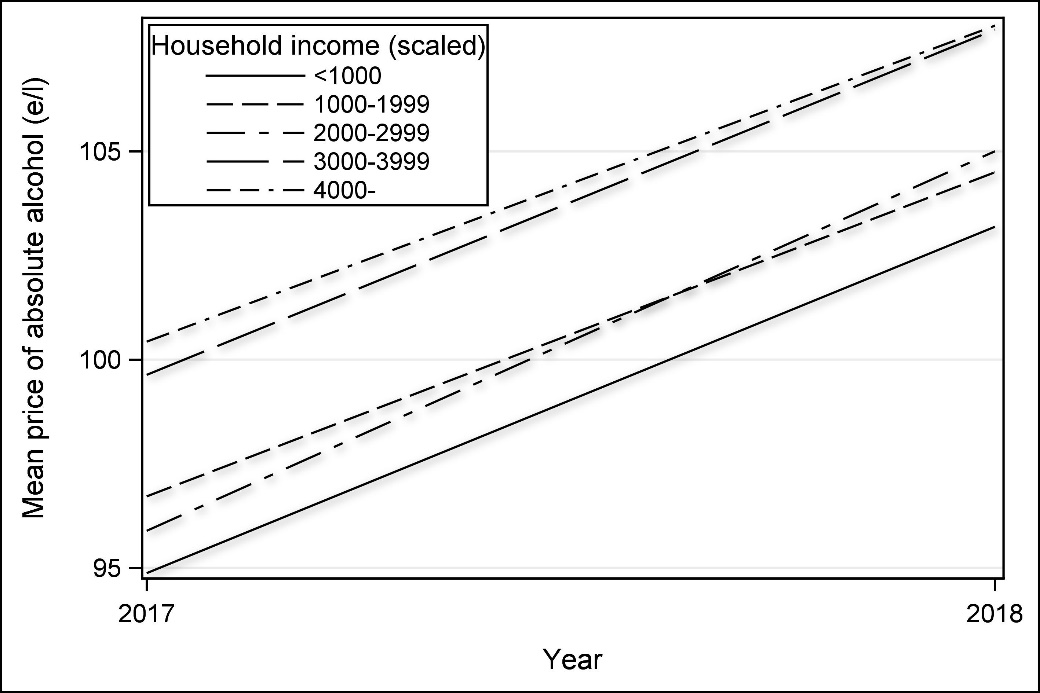


Figure S2. Mean price of alcoholic beverages (€/l absolute alcohol) by household income scaled according to household size (€/month) purchased by the loyalty card-holders of a large Finnish retail chain during the study years 2017 and 2018 (weighted to improve representativeness of the sample to the adult Finnish population).

Table S1. Expenditure on and price of alcohol purchased from grocery stores by household income (scaled for household size) in 2017 and 2018 (a year before and after the alcohol law reform) among loyalty card-holders of a large Finnish retail chain. The results are weighted to improve representativeness of the sample to the adult Finnish population.

|  | **Expenditure (€/year)** | | | | | | | | | | | | | | | | | | | | | | |
| --- | --- | --- | --- | --- | --- | --- | --- | --- | --- | --- | --- | --- | --- | --- | --- | --- | --- | --- | --- | --- | --- | --- | --- |
| **Household income (€/month)** | **Beer** | | | | | | **Cider** | | | | | | | **Ready-to-drink alcoholic beverages** | | | | | | **Total** | | | |
|  | **2017** | **2018** | | | **% Change** | | **2017** | | **2018** | | **% Change** | | | **2017** | | **2018** | | **% Change** | | **2017** | | **2018** | **% Change** |
| <1000 | 118.70 | 119.00 | | | 0.3 | | 16.30 | | 16.80 | | 3.1 | | | 13.60 | | 22.50 | | 65.4 | | 148.90 | | 158.50 | 6.5 |
| 1000-1999 | 107.70 | 108.50 | | | 0.7 | | 19.00 | | 19.40 | | 2.1 | | | 13.80 | | 25.50 | | 84.8 | | 140.50 | | 153.60 | 9.3 |
| 2000-2999 | 104.20 | 111.30 | | | 6.8 | | 15.80 | | 14.90 | | -5.7 | | | 11.90 | | 26.50 | | 122.7 | | 132.00 | | 152.80 | 15.8 |
| 3000-3999 | 117.30 | 127.70 | | | 8.9 | | 23.00 | | 20.70 | | -10.0 | | | 12.50 | | 33.30 | | 166.4 | | 152.90 | | 181.90 | 19.0 |
| ≥4000 | 114.70 | 125.40 | | | 9.3 | | 19.40 | | 18.60 | | -4.1 | | | 11.60 | | 30.00 | | 158.6 | | 145.80 | | 174.20 | 19.5 |
|  |  | |  |  | |  | |  | |  | |  |  | |  | |  | |  | |  | | |
|  | **Price (€/litre absolute alcohol)** | | | | | | | | | | | | | | | | | | | | | | |
| **Household income (€/month)** | **Beer** | | | | | | **Cider** | | | | | | | **Ready-to-drink alcoholic beverages** | | | | | | **Total** | | | |
|  | 2017 | 2018 | | | % Change | | 2017 | | 2018 | | % Change | | | 2017 | | 2018 | | % Change | | 2017 | | 2018 | % Change |
| <1000 | 72.80 | 79.20 | | | 8.8 | | 120.20 | | 123.70 | | 2.9 | | | 111.40 | | 128.70 | | 15.5 | | 86.40 | | 95.40 | 10.4 |
| 1000-1999 | 78.30 | 82.00 | | | 4.7 | | 126.80 | | 130.30 | | 2.8 | | | 111.10 | | 131.20 | | 18.1 | | 90.70 | | 98.30 | 8.4 |
| 2000-2999 | 79.90 | 86.80 | | | 8.6 | | 126.80 | | 131.10 | | 3.4 | | | 111.50 | | 131.40 | | 17.9 | | 90.80 | | 100.50 | 10.7 |
| 3000-3999 | 84.50 | 90.80 | | | 7.5 | | 132.10 | | 136.00 | | 3.0 | | | 114.00 | | 134.00 | | 17.5 | | 94.80 | | 103.50 | 9.2 |
| ≥4000 | 89.00 | 95.10 | | | 6.9 | | 134.00 | | 137.20 | | 2.4 | | | 113.90 | | 137.30 | | 20.5 | | 97.90 | | 105.60 | 7.9 |
